# Supplementary material for: Genetic markers of inflammation may not contribute to metabolic traits in Mexican children
Source: PeerJ. 2016 Jun 23;4:e2090. doi: 10.7717/peerj.2090 (PMC4924140; doi:10.7717/peerj.2090)
Supplement: Supplemental Information 1 [file peerj-04-2090-s001.docx]

**Supplementary Information**

**Genetic markers of inflammation do not contribute to metabolic traits in Mexican children**Neeti Vashi^1^, Carolina Stryjecki^1^, Jesus Peralta-Romero^2^, Fernando Suarez^2^, Jaime Gomez-Zamudio^2^, Ana I. Burguete-Garcia^3^, Miguel Cruz^2,*^, David Meyre^1,4,*^
^1^Department of Clinical Epidemiology and Biostatistics, McMaster University, Hamilton, ON, Canada

^2^Medical Research Unit in Biochemistry, Hospital de Especialidades, Centro Médico Nacional Siglo XXI del Instituto Mexicano del Seguro Social, Mexico City, Mexico

^3^Centro de investigación sobre enfermedades infecciosas. Instituto Nacional de Salud Pública. Cuernavaca, Morelos, Mexico

^4^Department of Pathology and Molecular Medicine, McMaster University, Hamilton, ON, Canada.

**Table of Contents**

Supplementary Table 1

Supplementary Figure 1

Supplementary Figure 2

**Supplementary Table 1. Description of the 6 SNPs studied.**

| Gene Name | Chromosomal Physical Location | SNP | Major Allele | Minor Allele | Genotype Count | Call Rate (%) | HWE P-value |
| --- | --- | --- | --- | --- | --- | --- | --- |
| *LEPR* | 1q31.3 | rs1137101 | A | G | 423 712 312 | 98.97 | 0.70 |
| *HNF1A* | 12q24.31 | rs7305618 | C | T | 1083 300 27 | 96.44 | 0.25 |
| *TNFA* | 6p21.3 | rs1800629 | G | A | 1343 118 1 | 100 | 0.33 |
| *IL-10* | 1q31-32 | rs1800896 | T | C | 791 527 84 | 95.90 | 0.76 |
|  |  | rs1800871 | G | A | 473 732 248 | 99.38 | 0.22 |
| *RETN* | 19p13.2 | rs1862513 | C | G | 1089 275 19 | 94.60 | 0.73 |

**Supplementary Figure 1. Power Calculation for the main effect of the SNPs on BMI.** Two-sided P-value of 0.05, 80% power.

**Supplementary Figure 2. Power calculation for the main effect of SNPs on BMI with Bonferroni correction.** Two sided P-value of 4.6x10^-4^, 80% power.
